# Vaticanol C, a resveratrol tetramer, activates PPARα and PPARβ/δ *in vitro *and *in vivo*

**DOI:** 10.1186/1743-7075-7-46

**Published:** 2010-05-27

**Authors:** Tomoko Tsukamoto, Rieko Nakata, Emi Tamura, Yukiko Kosuge, Aya Kariya, Michiko Katsukawa, Satoshi Mishima, Tetsuro Ito, Munekazu Iinuma, Yukihiro Akao, Yoshinori Nozawa, Yuji Arai, Shobu Namura, Hiroyasu Inoue

**Affiliations:** 1 Department of Food Science and Nutrition, Nara Women's University, Nara, 630-8506, Japan; 2 API research Center, API Co. Ltd., Gifu, 502-0071, Japan; 3 Laboratory of Pharmacognosy, Gifu Pharmaceutical University, Gifu 502-8585, Japan; 4 Gifu International Institute of Biotechnology, Gifu 504-0838, Japan; 5 Department of Bioscience, National Cardiovascular Center Research Institute, Suita, Osaka 565-8565, Japan; 6 Department of Neurobiology, Morehouse School of Medicine, 720 Westview Drive, SW, Atlanta, GA30310-1495, USA

## Abstract

**Background:**

Appropriate long-term drinking of red wine is associated with a reduced risk of cardiovascular disease. Resveratrol, a well-known SIRT1 activator is considered to be one of the beneficial components contained in red wine, and also developed as a drug candidate. We previously demonstrated that resveratrol protects brain against ischemic stroke in mice through a PPARα-dependent mechanism. Here we report the different effects of the oligomers of resveratrol.

**Methods:**

We evaluated the activation of PPARs by *ε*-viniferin, a resveratrol dimer, and vaticanol C, a resveratrol tetramer, in cell-based reporter assays using bovine arterial endothelial cells, as well as the activation of SIRT1. Moreover, we tested the metabolic action by administering vaticanol C with the high fat diet to wild-type and PPARα-knockout male mice for eight weeks.

**Results:**

We show that vaticanol C activates PPARα and PPARβ/δ in cell-based reporter assays, but does not activate SIRT1. *ε*-Viniferin shows a similar radical scavenging activity as resveratrol, but neither effects on PPARs and SIRT-1. Eight-week intake of vaticanol C with a high fat diet upregulates hepatic expression of PPARα-responsive genes such as cyp4a10, cyp4a14 and FABP1, and skeletal muscle expression of PPARβ/δ-responsive genes, such as UCP3 and PDK4 (pyruvate dehydrogenase kinase, isoform 4), in wild-type, but not PPARα-knockout mice.

**Conclusion:**

Vaticanol C, a resveratrol tetramer, activated PPARα and PPARβ/δ *in vitro *and *in vivo*. These findings indicate that activation of PPARα and PPARβ/δ by vaticanol C may be a novel mechanism, affording beneficial effects against lifestyle-related diseases.

## Background

Prevention of lifestyle-related diseases, such as cardiovascular disease, diabetes, and stroke, has become of worldwide interest. In such lifestyle-related matters, people pay attention not only to drugs, but also to the functionality of natural chemicals contained in food and drink, such as polyphenols and their polymers. In this context, resveratrol, a phytoalexin and antioxidant polyphenol included in red wine and various plants, is one of the most attractive compounds, and experimental data on resveratrol has been accumulating [1,2]. The polymers of resveratrol are also known phytoalexins and antioxidants. The resveratrol dimer, *ε*-viniferin, is a major phytoalexin in response to UV-C irradiation [3] and has been reported to be an antioxidant [4]. The resveratrol tetramer, vaticanol C, exhibited a more potent cytotoxic effect against human cultured cancer cells than resveratrol [5-7]. By screening 20 derivatives of resveratrol, vaticanol C was found to have the strongest suppressive activity of cell growth in colon cancer cell lines [6]. However, the molecular mechanisms for these actions remain to be determined.

We demonstrated that resveratrol activates peroxisome proliferator-activated receptor (PPAR) α and γ in cell-based reporter assays and protects the brain against ischemic stroke in mice through a PPARα-dependent mechanism [8]. PPARs are members of nuclear receptor family of ligand-dependent transcription factors [9]. The PPAR subfamily consists of three isotypes, PPARα, β/δ, and γ, which play various roles in lipid and carbohydrate metabolism, cell proliferation and differentiation, and inflammation and are considered to be molecular targets against lifestyle-related diseases [10,11]. For example, PPARα and γ agonists, such as fibrates and thiazolidine derivatives, are used to treat dyslipidemia and diabetes, respectively. Moreover, eicasapentaenoic acid, a natural ligand for PPARα, has been used as a hypolipidemic drug and has been reported to lower plasma and liver cholesterol levels in a PPARα-dependent manner [12]. Thus, we have focused on PPARs as possible molecular targets of resveratrol in preventing lifestyle-related diseases, while others have studied SIRT1, a NAD^+^-dependent protein deacetylase, and PGC-1α [13-17].

In the present study, we evaluated the activation of PPARs, antioxidant, and SIRT1-activator properties of resveratrol, ε-viniferin, and vaticanol C and found that vaticanol C activates PPARα and PPARβ/δ in cell-based reporter assays, but does not have antioxidant or SIRT1-activator properties. Moreover, 8-week intake of vaticanol C with a high fat (HF) diet upregulates hepatic expression of PPARα-responsive genes and skeletal muscle expression of PPARβ/δ-responsive genes in wild-type, but not PPARα-knockout mice. These findings indicate that activation of PPARα and PPARβ/δ by vaticanol C may provide beneficial effects against lifestyle-related diseases.

## Methods

### Reagents

ε-Viniferin, a resveratrol dimmer, and vaticanol C, a resveratrol tetramer, were isolated from the genus belonging to Vatica, Shorea, and Vateria (Dipterocarpaceae) as described previously [5,6]. A stock solution of these compounds was prepared in 10 mM dimethyl sulfoxide (DMSO), and was further diluted to the working concentration before use.

### Cell Culture

Bovine arterial endothelial cells (BAEC) [8,18] were grown in DMEM supplemented with 10% fetal calf serum and 50 μM 2-mercaptoethanol.

### RNA Extraction and Analysis

Total RNA was isolated using the acid guanidinium thiocyanate procedure, and was analyzed for gene expression via real-time quantitative RT-PCR (Mx3005, Stratagene) as described previously [18]. The primer pairs for genes in this study and their cycling conditions are shown in Additional file 1. Expression levels of each mRNA were normalized to those of 36B4 mRNA.

### Transcription Assays

In the case of activation assay for PPARα, BAEC was transfected with 0.15 μg of tk-PPREx3-Luc reporter plasmid, 0.15 μg of human PPARα expression vector pGS-hPPARα (Invitrogen, GeneStorm™ clone L02932) and 0.02 μg of pSV-βgal as described previously using Trans IT-LT-1 (Mirus) [19]. In the activation assay for PPARβ/δ and γ, their expression vectors pCMX-hNUCI (PPAR β/δ) and pCMX-hPPARγ1 were used for the transfection, instead of the human PPARα expression vector [18]. The transfected BAEC were harvested, lysed, and assayed for both luciferase and β-galactosidase activities. The results are represented as relative luciferase activities, which were normalized against the β-galactosidase standard. Transfection efficiency of BAEC is over 20% estimated by cotransfection of pEGFP-N1, an expression vector for Green Fluorescent Protein. pCMX-hNUCI and pCMX-hPPARγ1 were human PPARγ1 and PPARβ/δ expression vectors under control of a cytomegalovirus promoter [18]. In the positive control experiments, selective activators of PPARs, such as Wy-14643 (α), GW501516 (β/δ), and pioglitazone (γ), had PPARα, β/δ, and γ agonistic activities, respectively [18].

### DPPH Radical Scavenging and SIRT1 Activation Assays

Resveratrol and its derivatives were dissolved in DMSO to prepare samples at 500 μM. Twenty microliters of each sample, 80 μL of 0.1 M Tris HCl buffer (pH 7.4), and 100 μL of 500 μM 1,1-diphenyl-2-picryl-hydrazyl (DPPH) solution were mixed well and incubated at room temperature for 20 min, then the absorbance was recorded at 540 nm with a plate reader (Immuno mini NJ2300, Nalgene) as described [20]. Trolox (6-hydroxy-2,5,7,8-tetramethylchroman-2-carboxylic acid) was the reference antioxidant, used as a positive control, and each result is expressed as the equivalent per mole of trolox from the percentage decrease with respect to the negative control value. Data are presented as mean ± SD (*n *= 3).

The Fluor de Lys fluorescence assay for *in vitro *SIRT1 activity was performed by the SIRT1 activity assay kit (BIOMOL, catalog number AK-555), according to the manufacturer's instructions. Fluorescent intensity was measured using an Infinite F200 microplate fluorometer (TECAN). DMSO was used as a negative control and also as a solvent for resveratrol and its derivatives to a final concentration of 100 μM in the assay buffer.

### Animal Experiments

Male 12-week-old 129SV-strain (wild-type) and PPARα-knockout (129SV background) mice were obtained by Jackson laboratory (USA). These mice were housed in a room at 24 ± 2°C with a 12/12 h light-dark cycle, and fed a HF diet containing 60% energy as fat, supplemented with vaticanol C (0 and 0.04%) for 8 weeks. Diet and water were available *ad libitum*. After 8 weeks of feeding, the mice were sacrificed under diethyl ether anesthesia to obtain tissues. Liver and skeletal muscle were stored in RNAlater™ solution (Ambion, USA) at -30°C until gene expression analysis. These experimental procedures were approved by the Animal Care Committee of Nara Women's University.

### Statistical analyses

All results are expressed as means ± SD. Comparisons between groups were performed with the unpaired t test. Values were deemed to be statistically significantly different at p < 0.05.

## Results

### Activation of PPARs In Vitro by Resveratrol and Vaticanol C but not by ε-Viniferin

**Figure 1:**
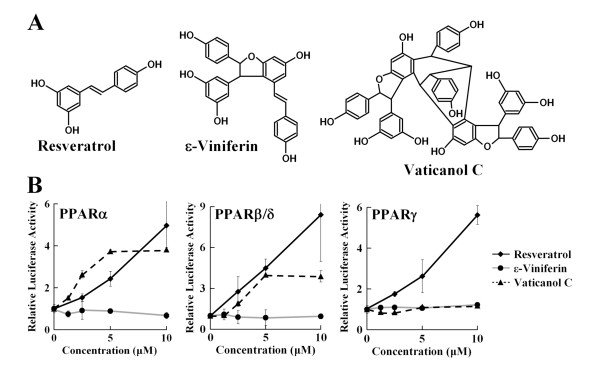
**Activation of PPARs by Resveratrol, ε-Viniferin and Vaticanol C**. (A) Structures of resveratrol (monomer), ε-viniferin (dimer) and vaticanol C (tetramer). (B) Activation of PPARα, β/δ, and γ was evaluated by transfection assays using BAEC with PPRE-luc together with GS-hPPARα, pCMX-NUC1 or pCMX-hPPARγ1, respectively. Results are presented as relative luciferase activities obtained by dividing the normalized luciferase activity from the reporter vector PPRE-luc. *, P < 0.05, **, P < 0.01 compared with the value of cells treated with ethanol (control) by unpaired t-test (*n *= 3).

**Figure 2:**
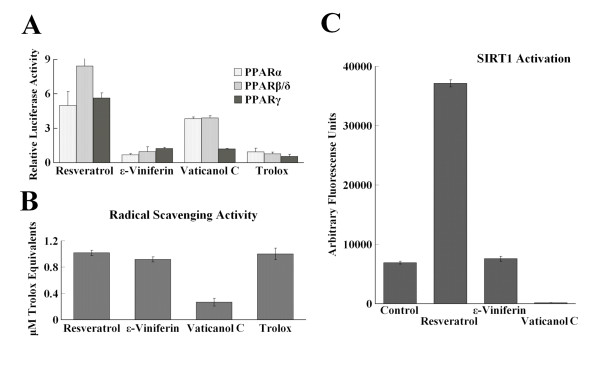
**Distinct Effects of Resveratrol, ε-Viniferin and Vaticanol C on Activation of PPARs, Radical Scavenging Activity and SIRT1 Activation**. (A) Activation of PPARα, β/δ, and γ was evaluated by cell-based transfection assays at a dose of 10 μM. Trolox, a standard antioxidant, was used as control. (B) Radical scavenging activity was evaluated by the DPPH method. Each result is expressed as the equivalent per mole of trolox from the percentage decrease with respect to the negative control values. (C) SIRT1 activation *in vitro *was evaluated by the Fluor de Lys fluorescence assay. DMSO was used as a negative control and also as a solvent for resveratrol and its derivatives to a final concentration of 100 μM in the assay buffer.

Resveratrol activated PPARα and γ in our cell-based assay using bovine arterial endothelial cells (BAEC) [8]. In this assay system, we also confirmed that selective activators of PPARs, such as Wy-14643 (α), GW501516 (β/δ), and pioglitazone (γ), had PPARα, β/δ, and γ agonistic activities, respectively [18]. Using this assay system, we evaluated the activation of PPARs by resveratrol, ε-viniferin, and vaticanol C (Figure 1A). Activation of PPARα and β/δ were observed by resveratrol and vaticanol C, whereas only resveratrol activated PPARγ (Figure 1B). These efficacies of resveratrol and vaticanol C on PPARα and β/δ were roughly similar to those of selective activators such as Wy-14643 and GW501516, respectively [18]. Remarkably, at lower concentrations, 1.25-5 μM, vaticanol C was a more potent activator for PPARα than resveratrol. On the other hand, ε-viniferin showed no agonistic activity for PPARs. At a concentration of 10 μM, resveratrol showed triple agonistic activities for α, β/δ and γ, whereas vaticanol C showed dual agonistic activities for α and β/δ Figure 2A). These properties of resveratrol and vaticanol C will provide beneficial effects against lifestyle-related disease since several drugs were reported to have similar properties.

### No Correlation with Resveratrol or its Derivatives between Activation of PPARs and Radical-Scavenging Activities

Next, we evaluated resveratrol and its derivatives for their radical-scavenging activity using DPPH, a stable free radical [20]. As shown in Figure 2B, resveratrol and ε-viniferin showed similar radical-scavenging activities, comparable to the positive control radical scavenger, trolox, a water-soluble derivative of vitamin E. On the other hand, vaticanol C showed no radical scavenging activity (Figure 2B), indicating that there was no correlation between agonistic activities for PPARs and radical-scavenging activity.

### No Activation of SIRT1 by Vaticanol C or ε-Viniferin

Because resveratrol has been reported to be an activator for SIRT1 [13], we evaluated the activation of SIRT1 by ε-viniferin and vaticanol C. As a positive control, resveratrol activated SIRT1; however, ε-viniferin and vaticanol C did not (Figure 2C). Rather, vaticanol C seemed to inhibit SIRT1 activity in this assay. Taken together, vaticanol C, a resveratrol tetramer, is a dual agonist for PPARα and β/δ, but not a radical scavenger and not a SIRT1 activator.

### Vaticanol C Administration Induces Expression of PPAR-dependent Genes

The metabolic actions of vaticanol C were then evaluated by administering the compound by food admixture (0 and 0.04%) to wild-type and PPARα-knockout male mice challenged with the HF diet for 8 weeks. During this experiment, there was no statistically significant difference in body weight between the eight groups.

**Figure 3:**
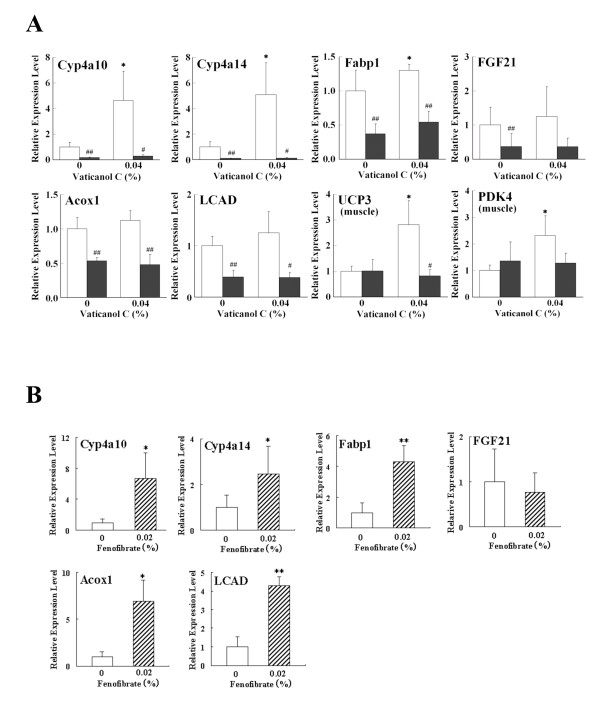
**Induction of PPAR-dependent Genes by Vaticanol C and Fenofibrate**. (A) mRNA levels of the indicated genes were measured by RT-qPCR in liver and skeletal muscle from WT (open column) and PPARα-knockout (closed column) mice fed a HF diet containing 60% energy as fat, supplemented with vaticanol C (0 and 0.04%) for 8 weeks. Results are presented as means ± S.D. (*n *= 4~7). * compared with the value of 0% vaticanol C (*, P < 0.05, **, P < 0.01). # compared with the value of the corresponding wild-type mice (#, p < 0.05, ##, p < 0.01).(B) mRNA levels of the indicated genes were measured by RT-qPCR in liver from WT fed a HF diet containing 60% energy as fat, supplemented with fenofibrate (0 and 0.02%) for 4 weeks. Results are presented as means ± S.D. (*n *= 4). * compared with the value of 0% fenofibrate (*, P < 0.05, **, P < 0.01).

We evaluated the effects of vaticanol C administration on the expression of PPAR-dependent genes in wild-type and PPARα-knockout mice. In the liver, expression of cyp4a10, cyp4a14 and fatty acid binding protein 1 (FABP1) was induced by 0.04% vaticanol C in the wild-type, but not in the knockout mice (Figure 3A). In the positive control, expression of these genes was also induced by 0.02% fenofibrate, a PPARα agonist, in the wild-type mice (Figure 3B). On the other hand, expression of FGF21, a PPARα-responsive protein, was not induced by 0.04% vaticanol C, and by 0.02% fenofibrate (Figure 3A, B), which may be due to feedback control of the PPARα-FGF21 endocrine pathway [21,22]. Expression of other PPARα-dependent genes, such as acyl-CoA oxidase (Acox1) and long-chain type acyl-CoA dehydrogenase (LCAD) was induced by 0.02% fenofibrate (Figure 3B) but not 0.04% vaticanol C (Figure 3A) in the wild-type mice, and was reduced in the knockout mice (Figure 3A). We also tested fenofibrate in PPARα-knockout mice on high fat diet (n = 2) or on normal fat diet (n = 4); however, no induction of PPARα-responsive genes was observed (data not shown), which is similar to the results of vaticanol C with a HF diet. The discrepancies between fenofibrate and vaticanol C were unknown, but may be involved in other regulating factors for gene expression. In this point, we found that 0.04% resveratrol treatment with a normal diet, but not with a HF diet, showed induced expression of Acox1 and LCAD (manuscript in preparation), which are not induced by vaticanol C treatment with a HF diet (Figure 3A). In skeletal muscles, expression of PPARβ/δ-dependent genes, such as UCP3 and PDK4 [23,24], was induced by vaticanol C in wild-type, but not in knockout mice (Figure 3A).

## Discussion

Resveratrol is known to be a SIRT1-activator. However, various effects of resveratrol are not explained by activation of SIRT1 alone. We found that resveratrol is an activator not only for PPARα and γ [8], but also for PPARβ/δ (Figure 1B) in this report. Moreover, vaticanol C, a resveratrol tetramer, is an activator for PPARα and β/δ in cell-based reporter assays (Figure 1B), which is confirmed by induced expression of PPAR-responsive genes in wild-type, but not PPARα-knockout, mice (Figure 3A). At lower concentrations of 1.25-5 μM, vaticanol C showed higher PPARα-agonistic activity than resveratrol. However, at 10 μM, resveratrol showed higher activity than vaticanol C (Figure 1A), and this activity of resveratrol was dose-dependent, at less than 40 μM (data not shown). Previously, we found that expression of COX-2, an inducible key enzyme for prostaglandin synthesis, was regulated by 15d-PGJ_2_, a natural ligand for PPARγ [19]. Interestingly, vaticanol C as well as resveratrol [25] suppressed the expression of COX-2 in several kinds of tumor cell lines (data not shown) whereas vaticanol C is not an activator for PPARγ.

Vaticanol C did not activate SIRT1 whereas resveratrol activated SIRT1 by the assay utilizing a fluorophore-containing peptide (Figure 2C). Recent report showed that this assay was not suitable to direct measurement of the SIRT1 activity and that resveratrol did not activate SIRT1 in the assay without the fluorophore-containing peptide [26]. Therefore, by the different assay system, we may confirm that vaticanol C is not activator for SIRT1. Taken together, vaticanol C is a dual activator for PPARα and β/δ, whereas resveratrol is a triple activator for PPARα, β/δ and γ, indicating that vaticanol C is thought to be differently effective on lifestyle-related diseases, compared with resveratrol.

A resveratrol dimer, ε-viniferin has radical-scavenging activity, but does not have agonistic activity for PPARs or SIRT1, which differs from resveratrol and vaticanol C (Figure 2). These differences indicate the importance of the structure of these chemicals. Concerning the binding pocket of PPARα, the molecular sizes of resveratrol and vaticanol C are smaller and larger than those of synthetic PPAR agonists such as fenofibrate, respectively. Resveratrol but not vaticanol C activates PPAR γ, indicating the importance of the structure of these molecules in the binding pocket of PPARs. It was reported that a single amino acid, which was Tyr in PPARα and His in PPARγ, imparted subtype selectivity for both thiazolidinedione and nonthiazolidinedione ligands [27]. We need more experimental data to discuss about these points including the possibility of indirect activation of PPARs by resveratrol and vaticanol C.

There are distinct expression patterns of PPAR-responsive genes in the liver and skeletal muscle, indicating that PPAR-responsive genes are controlled in a tissue-specific manner (Figure 3A). In this context, especially in skeletal muscles, vaticanol C upregulates expression of PPARβ/δ-responsive genes, such as UCP3 and PDK4, in the wild-type, but not PPARα-knockout mice, indicating that PPARα is also involved in the expression of PPARβ/δ-responsive genes.

Resveratrol has been reported to prevent body weight gain with a HF diet [15]. No such effect on body weight was observed after vaticanol C treatment. We found that 129SV mice used in our study showed milder body weight gain with a HF diet, versus the C57/black strain mice used in several reports. This difference on body weight gain will be due to the different strains between 129SV and C57/black. These genetic differences are also usual common in human genomes. To address these questions, we are studying on the molecular mechanism involved in the physiological differences between 129SV and C57/black mice.

## Conclusion

Vaticanol C, a resveratrol tetramer, activates PPARα and PPARβ/δ in cell-based reporter assays, but does not activates SIRT1. Eight-week intake of vaticanol C with a HF diet upregulates hepatic expression of PPARα-responsive genes and skeletal muscle expression of PPARβ/δ-responsive genes in wild-type but not PPARα-knockout mice. These findings indicate that activation of PPARα and PPARβ/δ by vaticanol C is a novel mechanism that may afford beneficial effects against cardiovascular disease.

## Competing interests

The authors declare that they have no competing interests.

## Authors' contributions

TT, RN, ET, YK, AK, MK and YA carried out the experiments and analyzed the data. SM, TI, MI, YA and YN prepared ε-viniferin and vaticanol C. RN, SN and HI designed the experiments and wrote the manuscript. All authors read and approved the final manuscript.

## Supplementary Material

Additional file 1 **Supplementary Table 1**. Primer pairs for genes and their cycling conditions are shown. Click here for file
