# Supplementary material for: Vaticanol C, a resveratrol tetramer, activates PPARα and PPARβ/δ in vitro and in vivo
Source: Nutr Metab (Lond). 2010 May 27;7:46. doi: 10.1186/1743-7075-7-46 (PMC2882917; doi:10.1186/1743-7075-7-46)
Supplement: Additional file 1 — Supplementary Table 1. Primer pairs for genes and their cycling conditions are shown. [file 1743-7075-7-46-S1.PDF]

Supplementary Table 1

Gene	GenBank Accession	Primer Sequence Forward (F), Reverse (R)	Product size (bp)	Annealing Temperature
cytochrome P450, family 4, subfamily a, polypeptide 10 [Cyp4a10]	AB018421	F: 1387-ccaggaactgcattgggaaa<br>R: 1487-gaccctggtaggatctggca	101	62
cytochrome P450, family 4, subfamily a, polypeptide 14 [Cyp4a14]	NM_007822	F: 1202-acctgtcaccttcccagatg<br>R: 1406-agcaaactgtttcccaatgc	205	65
fatty acid binding protein 1, liver [Fabp1]	NM_017399	F: 54-aagtaccaattgcagagccagga<br>R: 230-ggtgaactcattgcggacca	177	60
fibroblast growth factor 21 [FGF21]	NM_020013	F: 480-cctctaggtttctttgccaacag<br>R: 555-aagctgcaggcctcaggat	76	67
acyl-Coenzyme A oxidase 1, palmitoyl [Acox1]	AF006688	F: 670-gggagtgctacgggttacatg<br>R: 760-ccgatatccccaacagtgatg	91	62
acyl-Coenzyme A dehydrogenase, long-chain [LCAD]	NM_007381	F: 1075-cagttgcatgaaaccaaacg<br>R: 1299-gacgatctgtcttgcgatca	225	65
pyruvate dehydrogenase kinase, isoenzyme 4 [PDK4]	NM_013743	F: 956-cgtcttgggaaaagaagacct<br>R: 1044-tgtaactaaagaggcggtcagtaa	89	65
uncoupling protein 3 (mitochondrial, proton carrier) [UCP3]	NM_009464	F: 767-tgctgagatggtgacctacg<br>R: 928-gcgttcatgtatcgggtctt	162	65
ribosomal protein, large, P0 [36B4]	NM_007475	F: 92-ggcgacctggaagtccaact<br>R: 204-ggatctgctgcatctgcttg	113	65
